# P-1633. Disparities in the Use of nirmatrelvir/ritonavir for COVID-19: A Retrospective Cohort Study

**DOI:** 10.1093/ofid/ofaf695.1809

**Published:** 2026-01-11

**Authors:** Maureen Campion, Majd Alsoubani, Gabriela Andujar Vazquez

**Affiliations:** Tufts Medical Center, Boston, Massachusetts; Tufts Medical Center, Boston, Massachusetts; Tufts Medical Center, Boston, Massachusetts

## Abstract

**Background:**

COVID-19 has caused significant morbidity and mortality which disproportionately impacted racial and ethnic minorities. The introduction of nirmatrelvir/ritonavir (n/r) has introduced an oral option for treatment, but its use was limited by drug-drug interactions. In this study, we aim to evaluate the factors associated with the prescription of nirmatrelvir/ritonavir (n/r) compared to other COVID-19 antivirals in a healthcare system in Eastern Massachusetts.

**Table 1:**
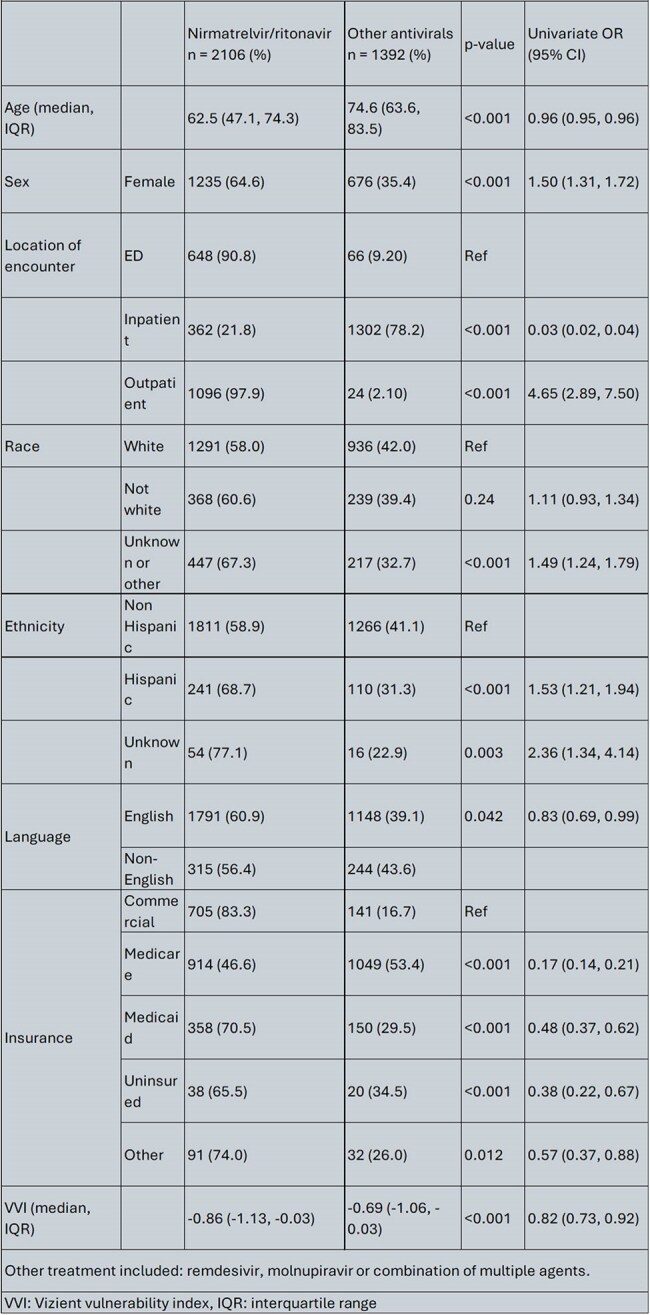
Baseline demographic and clinical characteristics of patients who received nirmatrelvir/ritonavir compared to other treatments

**Table 2:**
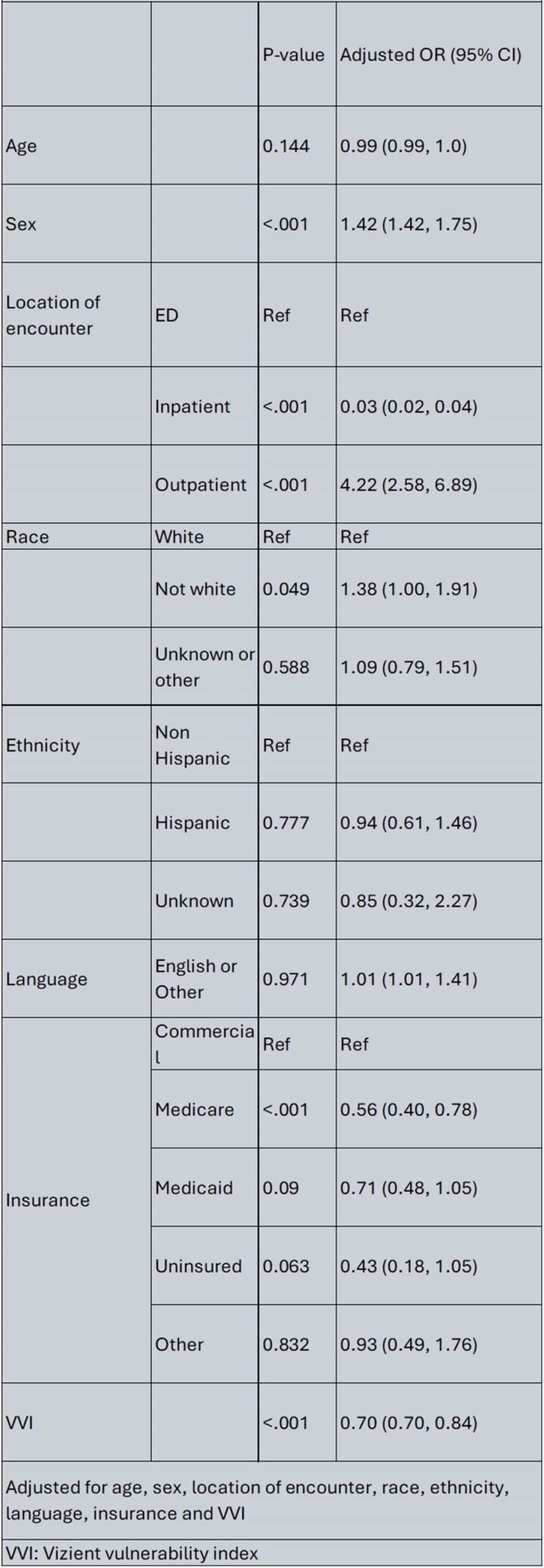
Adjusted logistic regression model of patients who received nirmatrelvir/ritonavir compared to other treatments

**Methods:**

This is a retrospective study including adult patients with a COVID-19 diagnosis. The Vizient Clinical Database, which captures patient-level data, was used to identify encounters between Oct/2022 and Jul/2024. The primary study outcome was evaluating patients who received COVID-19 antivirals with molnupiravir, remdesivir or combination of multiple agents compared to those who received n/r. Demographic data including Vizent vulnerability index (VVI) were collected. VVI score ranges between -3 to 3, higher values indicates increased vulnerability. Patient characteristics by treatment group were presented as counts and percentages for categorical variables and medians with interquartile ranges for continuous variables. The primary analysis was a logistic regression model evaluating factors associated with n/r prescription.

**Results:**

We identified 3498 who received COVID-19 antivirals. Most patients received n/r for treatment (n = 2106, 60.2%). Patients’ characteristics are outlined in Table 1. In the multivariate model female sex, outpatient encounters, higher VVI (more socially vulnerable) and non-White race remained significantly associated with increased odds of being prescribed n/r (Table 2). Inpatient encounter and Medicare coverage were associated with reduced odds of getting n/r as compared to patients seen in the ED.

**Conclusion:**

Prescriptions for n/r is different across the healthcare system. N/r was found to be prescribed more in the outpatient and ED setting which is unsurprising based upon its oral dosage form. However, patients with lower socioeconomic status based upon the VVI and non-White were more likely to be prescribed n/r. These findings highlight demographic and socioeconomic differences in the prescribing patterns of COVID-19 antiviral therapies.

**Disclosures:**

All Authors: No reported disclosures

